# Nationwide emergence and spread of highly virulent PRRSV-2 mutants in Korea

**DOI:** 10.1186/s40813-025-00470-5

**Published:** 2025-11-11

**Authors:** Seung-Chai Kim, Sang Chul Kang, Hwan-Ju Kim, Jonghyun Park, Hye-Ryung Kim, Choi-Kyu Park, Won-Il Kim

**Affiliations:** 1 College of Veterinary Medicine, Jeonbuk National University, 79 Gobong-ro, Iksan, 54596 Republic of Korea; 2 Optipharm Co. Ltd, Cheongju, 28158 Republic of Korea; 3 DIVA Bio Inc, Iksan, 54531 Republic of Korea; 4 College of Veterinary Medicine & Animal Disease Intervention Center, Kyungpook National University, Daegu, 41566 Republic of Korea

**Keywords:** PRRSV-2, ORF5, Lineage classification, NADC34-like, Sow mortality, Post-weaning mortality, Phylogenetics, Korea

## Abstract

Porcine reproductive and respiratory syndrome virus type 2 (PRRSV-2) continues to pose a major threat to global swine health. In South Korea, the co-circulation of endemic strains and repeated introductions of foreign variants has led to a complex viral landscape. This study analyzed 907 non-redundant ORF5 sequences from Korean field isolates (2018–2024), derived from 1,305 compiled sequences after removing near-duplicates at 99.9% identity using CD-HIT, together with 996 global reference ORF5 sequences, to refine phylogenetic classification and trace recent epidemiological patterns. A previously undefined Korean-specific clade (formerly referred to as Lineage KOR B or LKB) was proposed as Lineage 12 (L12), based on > 15% nucleotide divergence from existing lineages. In addition, we identified localized diversification within Lineage 1 sublineages (L1A and L1C), suggesting regional adaptation after foreign introduction. The most striking observation was the rapid emergence and spread of NADC34-like PRRSV (L1A). First detected in 2022, L1A rose from 11.7% to 23.3% of relative positive rates by 2024 and disseminated across major swine-producing provinces. A representative case in 2023 was associated with severe reproductive failure including severe abortion and high mortality in newborn piglets. Notably, the outbreak also demonstrated unusually high sow mortality (~ 10%), indicating high virulence of Korean NADC34-like PRRSV strains. Postmortem examinations from other outbreaks revealed consistent systemic lesions in deceased sow or gilts, indicating a reproducible pathological signature of Korean NADC34-like strains in old-aged pigs. These findings highlight the evolving virulence of PRRSV-2 in Korea and underscore the urgent need for updated lineage surveillance, strain-specific vaccines, and enhanced transboundary disease monitoring frameworks.

**Supplementary Information:**

The online version contains supplementary material available at 10.1186/s40813-025-00470-5.

## Introduction

Porcine reproductive and respiratory syndrome virus (PRRSV) is regarded as one of the most economically devastating pathogens affecting the global swine industry, with annual losses in the United States alone estimated at approximately $664 million [1]. The causative viral agent, PRRS virus (PRRSV) is a small, enveloped, single-stranded positive-sense RNA virus with a genome of approximately 15 kb in length, which belongs to the order *Nidovirales*, family *Arteriviridae*. PRRSV is classified into two species: *Betaarterivirus suid 1* (PRRSV-1, European type, prototype Lelystad virus) and *Betaarterivirus suid 2* (PRRSV-2, North American type, prototype VR2332) (ICTV2021). PRRSV primarily infects porcine alveolar macrophages (PAMs) in the lungs [2, 3], but additional macrophage subsets, such as pulmonary intravascular macrophages (PIMs) in the lung, may be preferentially targeted by highly virulent strains [4]. Clinical outcomes vary depending on host age and physiological status: in weaned piglets respiratory distress accompanied by immunosuppression predominates [5, 6], while in pregnant sows the major manifestations are abortion, stillbirth, premature farrowing and the birth of weak-born piglets induced by the infection during late gestation [5, 7–9]. These reproductive failures substantially increase preweaning mortality and productivity losses [10–14].

Among the at least 11 open reading frames (ORFs) encoded by PRRSV, the ORF5 gene, encoding the major envelope glycoprotein GP5, plays a key role in viral assembly, infectivity, and the induction of virus-neutralizing antibodies [15–17]. Notably, ORF5 exhibits the highest level of genetic variability among PRRSV structural genes, making it a widely used marker for molecular epidemiology and phylogenetic classification, although it only comprises 5% of the genome. Based on sequence analysis of ORF5, PRRSV-1 has recently been classified into four lineages and 18 sublineages [18], while PRRSV-2 has been refined into eleven lineages with 21 sublineages, including L1A–L1F, L1H–L1J, L5A–L5B, L8A–L8E, and L9A–L9E [19]. PRRSV-2 lineages show genetic and antigenic heterogeneity that can influence pathogenicity and immunogenicity [20, 21]. However, establishing a direct relationship between lineage designation and virulence is problematic, as virulence is a multigenic trait and not determined solely by ORF5. Highly virulent strains often exhibit enhanced replication capacity [20, 21], a feature more commonly associated with non-structural proteins (NSPs) [22, 23], and recombination can give rise to variants that retain virulence traits while belonging to a different ORF5 lineage [24–26]. Nevertheless, the high variability of ORF5 and its broad availability in diagnostic datasets have ensured that ORF5-based classification remains an effective tool for monitoring epidemic dynamics and tracing the spread of emerging variants in the field [18, 19].

In recent years, sublineages within lineage 1 (L1), particularly L1C (e.g., strain NADC30; GenBank accession number JN654459) and L1A (e.g., strain NADC34; MF326985), which originated in North America, have spread globally across swine-producing regions, including the United States, China, and beyond [20, 27–31]. Among them, the NADC30-like (L1C) viruses, first identified in the U.S. in 2008 and characterized by discontinuous 131 amino acid deletion in the NSP2 region [32], have induced multiple outbreaks and frequently detected as recombinants with other lineages [24–26]. The NADC34-like (L1A) virus was first identified in the U.S. in 2014 and has since been associated with outbreaks marked by “abortion storms” in sow herds and high neonatal mortality [33, 34]. Characterized by a distinctive 100-amino acid deletion in the NSP2 region, L1A strains have subsequently been reported in Peru (2015) [35], China (2017) [36], Korea (2022) [37], and Japan (2022) [38], raising concerns about their emerging global impact.

South Korea, situated on the Korean Peninsula in East Asia, has historically exhibited a unique PRRSV-2 phylogenetic profile distinct from that of other major swine-producing countries. Until the early 2010s, the majority of circulating PRRSV-2 strains in Korea were either derived from the Ingelvac^®^ modified live virus (MLV) vaccine strains (L5A) or belonged to Korean-specific lineages, namely KOR A (LKA), B (LKB), and C (LKC) [39, 40], which were recently reclassified as L11, undefined, and L1J, respectively [19]. These patterns suggest a regionally distinct evolutionary profile, possibly shaped by local ecological and epidemiological pressures common in animal agriculture settings [41]. However, since 2014, the increasing importation of breeding pigs from North America has facilitated the introduction of novel lineage 1 viruses, particularly NADC30-like strains (L1C), which rapidly became the dominant lineage by 2020 [32, 40]. In 2022, the first detection of NADC34-like PRRSV (L1A) was reported in Korea, associated with an evident pattern of vertical transmission within the herd [37]. This finding raises the possibility of yet another epidemic lineage establishing itself within the Korean swine population. Notably, the current epidemiological trends in Korea appear to parallel those observed in the United States [27, 28], and China [29, 31], where newly emerging lineage 1 subtypes are displacing previously dominant strains. Therefore, the need to characterize the evolutionary dynamics and clinical impact of Korean NADC34-like PRRSV has become increasingly urgent.

In this study, we investigated the phylogenetic relationships of ORF5 genes from Korean PRRSV-2 isolates collected up to 2024, alongside globally circulating strains, to elucidate the current molecular epidemiology of PRRSV-2 in South Korea. Additionally, we report the first well-documented outbreak case of NADC34-like PRRSV (L1A) in Korea, which occurred in May 2023, and present associated clinical and pathological findings from this and subsequent cases. By integrating phylogenetic analysis with field-level epidemiological data, our study not only deepens the understanding of the emergence and local spread of a potentially epidemic lineage, but also offers broader insights into the transboundary evolution of PRRSV-2 and its implications for global swine health management.

## Materials & methods

### ORF5 sequence dataset of Korean PRRSV-2

From 2021 to 2024, a total of 835 PRRSV-2 ORF5 sequences were obtained from clinical specimens, including lung, lymph node, and serum samples, submitted to the Jeonbuk National University Veterinary Diagnostic Center (JBNU-VDC). The detailed procedures for tissue processing, RNA extraction, primer usage, reverse transcription polymerase chain reaction (RT-PCR), and ORF5 sequencing followed protocols described previously. The sequences were deposited in GenBank under accession numbers OQ848759–OQ848988 (2021–2022) and PV425456–PV425876 (2023–2024).

In addition, 239 PRRSV-2 ORF5 sequences collected between 2018 and 2021 from clinical samples processed in Kyungpook National University (KNU) were incorporated into the Korean PRRSV-2 sequence dataset (GenBank accession numbers: ON420480–ON420718). Further Korean PRRSV-2 ORF5 sequences available in GenBank as of 2024 (*n* = 231) were also included. However, no publicly available ORF5 sequences from Korea were identified for the years 2023 and onward.

To minimize redundancy and improve the resolution of subsequent phylogenetic analyses, we combined a total of 1,305 Korean PRRSV-2 ORF5 sequences, consisting of 835 sequences from JBNU-VDC (2021–2024), 239 sequences from KNU (2018–2021), and 231 sequences retrieved from GenBank (2018–2022). These 1,305 Korean PRRSV-2 ORF5 sequences were subjected to subsampling using CD-HIT v4.8.1 [42] with a 99.9% sequence identity threshold. This step aimed to eliminate duplicate sequences from identical farms or outbreak events. The final dataset comprised 907 non-redundant Korean PRRSV-2 ORF5 sequences collected from 2018 to 2024, and was used for downstream analysis. The annual and regional distribution of these sequences is summarized in Supplementary Table 1.

### Collection of reference sequence dataset and data processing

A global PRRSV-2 ORF5 reference dataset was constructed based on the lineage-defining sequences proposed in the refined phylogenetic system by Yim-Im et al. [19]. These sequences were subsampled using CD-HIT at a 99% similarity cutoff. Lineage 7 (L7, *n* = 24) sequences were manually curated and added, resulting in 767 reference sequences.

To enhance coverage, Chinese lineage 1 (L1) viruses from 2018 to 2022 were retrieved and subsampled using a 95% similarity threshold (*n* = 201). Korean representative strains (*n* = 22) [43] along with whole-genome sequenced Korean L1A strains (*n* = 7), were also included. The final reference panel comprised 996 ORF5 sequences representing global PRRSV-2 diversity.

To evaluate the potential influence of recombination on phylogenetic inference, all 1,903 PRRSV-2 ORF5 sequences analyzed in this study were tested using RDP4 (v4.101) by seven detection algorithms (RDP, GENECONV, BootScan, MaxChi, Chimaera, SiScan, and 3Seq), with Bonferroni correction. Detection using all seven methods implemented in RDP4 was taken as significant evidence for recombination.

### Phylogenetic analysis

Multiple sequence alignment (MSA) of 1,903 PRRSV-2 ORF5 sequences was performed using the Fast Fourier Transform algorithm with a normalized similarity matrix (FFT-NS-2) implemented in MAFFT v7.520 [44]. Alignments were manually inspected and refined using AliView [45]. Maximum likelihood (ML) phylogenetic trees were constructed using RAxML-NG [46] under the general time-reversible model with FreeRate heterogeneity across sites (GTR + F + R10), with 1,000 bootstrap replicates to assess branch support. Genetic lineages and sublineages were classified based on the recently proposed refined phylogenetic system by Yim-Im et al. [19]. Resulting ML trees were visualized using the *ggtree* package in R software [47], and pairwise nucleotide identities were calculated using MEGA X [48].

### Nationwide surveillance of PRRSV-2 epidemiology in Korea

To characterize recent epidemiological trends of PRRSV-2 in Korea, we analyzed the temporal dynamics of circulating lineages detected in field cases between 2018 and 2024. Lineage classification was based on ORF5-based phylogenetic inference, and yearly detection frequencies were visualized using R software to monitor shifts in dominant lineages.

Following the first detection of NADC34-like PRRSV (L1A) in 2022 [37], a substantial increase in L1A-positive field cases was observed in 2023 and 2024. To assess its spatial dissemination, sample collection sites of NADC34-like PRRSV cases (2022–2024) were geographically visualized. Annual spatial distributions were visualized using provincial maps, overlaid with pig population data retrieved from the Korean Statistical Information Service (KOSIS) (https://kosis.kr/eng/), as previously described [37].

### Case presentation: farm information and L1A PRRSV outbreak history

Clinical and production data were collected from a commercial farrow-to-nursery pig farm (Farm A) housing approximately 3,000 sows, located in Chungcheongnam-do (CN) province, Republic of Korea. Notably, this is the same province where NADC34-like PRRSV was first reported in Korea [37]. The farm operated on a weekly batch farrowing system, with approximately 130 sows farrowing per week. Piglets were weaned at around 24 days of age and transferred in batches of ~ 1,500 to nursery facilities equipped with fully slatted floors and negative-pressure ventilation, maintaining ambient temperatures of 25–30 °C depending on piglet weight. After the nursery phase, piglets of approximately 25–30 kg were either transferred to off-site fattening units or sold to other finishing farms.

The farm maintained a strict biosecurity protocol under the guidance of attending veterinarians. In 2022, pre-outbreak productivity indicators included a farrowing rate of 82.7%, total piglets born per litter (TB) of 13.5, piglets born alive (BA) of 12.3, weaned piglets per litter (WP) of 11.5, and an annual weaned per sow per year (PSY) of 25.6. Prior to the outbreak, the herd was routinely vaccinated with a PRRSV-2 MLV vaccine (Ingelvac^®^ PRRS MLV, Boehringer Ingelheim, Germany). On 27 May, 2023, clinical signs including reduced feed intake and high fever (> 40 °C) were observed in pregnant sows. Within a week, the farm experienced a surge in abortion events and sow mortalities.

To further characterize the outbreak strain, serum samples collected from an affected sow and a suckling piglet during the outbreak were submitted to the JBNU-VDC for virus isolation and whole-genome sequencing. Virus isolation was performed using primary porcine alveolar macrophages (PAMs) derived from PRRSV-negative four-week-old weaned piglets, as previously described [37]. Complete genome sequences were obtained using a sequence-independent single primer amplification (SISPA) next-generation sequencing (NGS) approach, implemented on the Illumina iSeq100 platform with 150 bp paired-end reads. De novo assembly was performed using an in-house bioinformatics pipeline, also described previously [43]. The complete genomes of viruses isolated from the sow and piglet samples were 100% identical. The isolate was designated JBNU-23-N05, and the genome sequence was deposited in GenBank under accession number PV459697.

### Assessment of the production impact and clinical pattern due to the outbreak

Production data encompassing breeding and nursery performance were provided by farm personnel and veterinarians, based on standard industry metrics and definitions [49]. To evaluate the impact of the NADC34-like PRRSV outbreak, performance data were analyzed over two consecutive 19-week periods: before and after the clinical onset of PRRSV symptoms in the sow herd.

The dataset included breeding outcomes, parity distributions, return-to-estrus rate (RR), total piglets born (TB), piglets born alive (BA), weaned piglets (WP), as well as weekly records of sow deaths, abortions, and piglet mortality during both pre- and post-weaning stages. Temporal trends were visualized, and differences assessed using GraphPad Prism v10.0.3 (GraphPad Software, USA). Correlation between sow deaths and abortion incidence was evaluated via simple linear regression.

Odds ratios (ORs) with 95% confidence intervals were calculated to compare pre- and post-outbreak risk for major outcomes. Statistical significance was determined using Fisher’s exact test, with p-values interpreted as follows: *p* < 0.05 (*), < 0.01 (**), < 0.001 (***) and < 0.0001 (****).

### Histopathological examinations of NADC34-like PRRSV-affected sows from outbreak cases

To assess the pathological findings of NADC34-like PRRSV infection in the field, postmortem tissue samples were collected and subjected to Animal Clinical Evaluation Center, Optipharm Co. Ltd. from sows that experienced clinical reproductive failure during confirmed NADC34-like PRRSV outbreak cases in March 2023 (Farm B, located in Gyeonggi-do province) and March 2024 (Farm C, located in Chungcheongbuk-do province). Farm B is a farrow-to-nursery unit housing approximately 2000 sows, and Farm C is a farrow-to-finish unit with about 400 sows. Pig herds in both farms were routinely vaccinated with PRRSV-2 MLV vaccine prior to the outbreaks. Necropsies for both cases were performed on-site immediately after death, and other swine pathogens (including classical swine fever virus, *Acinobacillus suis*, *Erysipelothrix rhusiopathiae*, and *Salmonella* spp.) were excluded by PCR/RT-PCR. The collected tissues were further subjected to ORF5 sequencing, and the sequences were deposited in GenBank under accession number PV425837 (Farm B) and PV425849 (Farm C), respectively.

For histopathological examination, each sow case was observed in detail during necropsy to determine characteristic macroscopic features in internal organs. After necropsy, all major organs in the thoracic and abdominal cavities were selectively excised including macroscopic lesions and fixed in 10% neutral-buffered formalin (NBF). Following fixation, tissue samples were trimmed to include lesions, routinely processed, and embedded in paraffin. Paraffin blocks were sectioned at approximately 3–4 μm thickness and stained with hematoxylin and eosin (H&E) for light microscopy examination. Tissue slides were examined under a light microscope (BX53, Olympus, Japan) to assess histopathological changes.

To detect PRRSV antigens in the organs, replicate sections of internal organs used for immunohistochemistry (IHC) with anti-PRRSV monoclonal antibody at 1:1,000 dilution (9041, Median Diagnostics, Republic of Korea) after digestion with 0.05% protease XIV (Sigma, USA) for antigen retrieval. Dako REAL™ EnVision™ Detection System, Peroxidase/DAB + Rabbit/Mouse (K 5007, Dako, Denmark) was used for IHC according to the manufacturer’s instructions.

## Results

### Phylogenetic classification and sublineage structure of PRRSV-2 ORF5 sequences in Korea

**Fig. 1:**
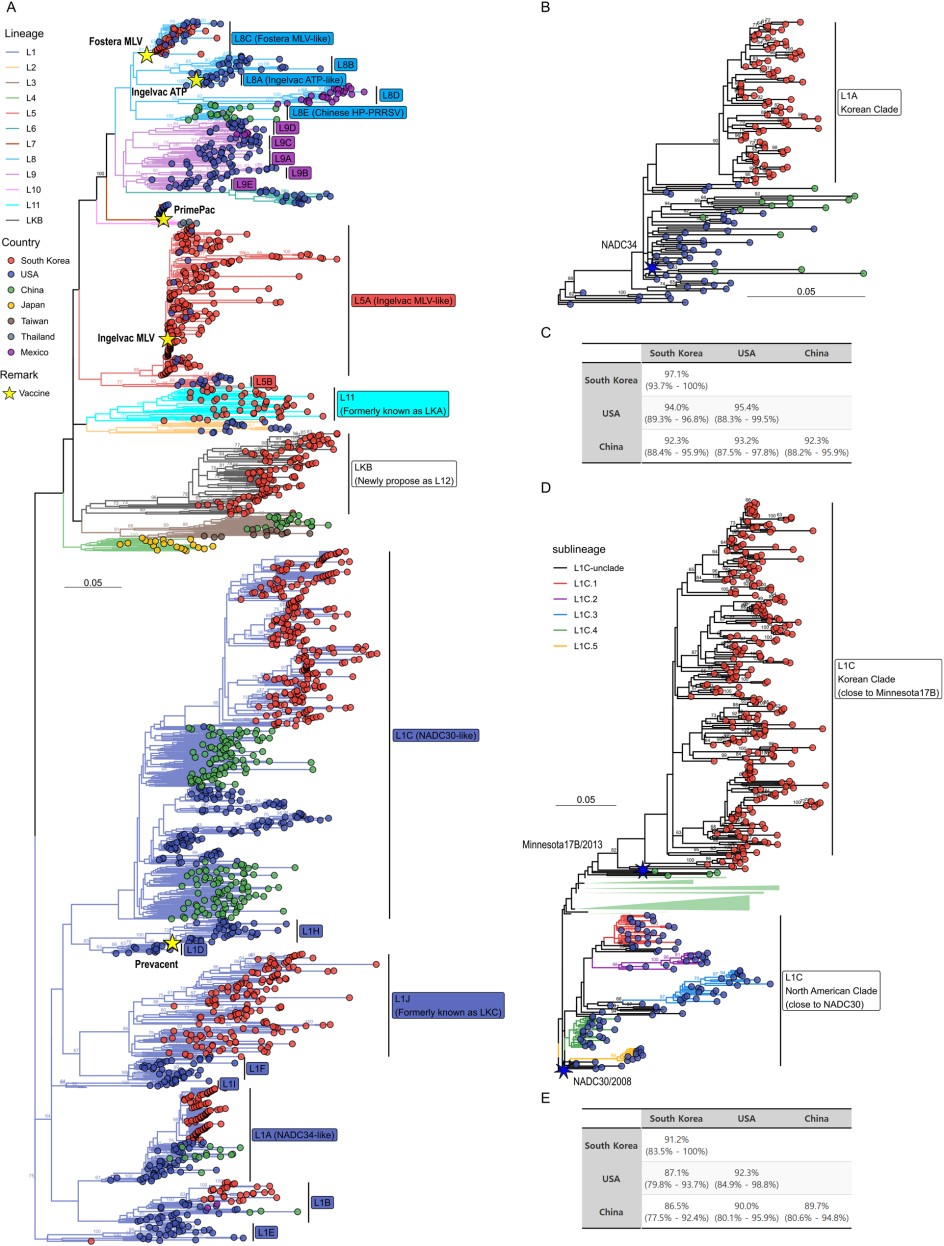
Phylogenetic classification and sequence homology of PRRSV-2 ORF5 strains in Korea. (**A**) Maximum likelihood (ML) phylogenetic tree of 907 non-redundant Korean PRRSV-2 ORF5 sequences (2018–2024) and 996 global reference PRRSV-2 ORF5, constructed using the GTR + F + R10 model with 1,000 bootstrap replicates. Sequences were classified according to the refined system proposed by Yim-Im et al. [19]. (**B**) Sublineage-specific ML phylogenetic tree of L1A PRRSV-2 strains, highlighting the genetic divergence between Korean, U.S., and Chinese L1A sequences. (**C**) Pairwise ORF5 nucleotide identity (%) between Korean, U.S., and Chinese L1A sequences, as well as within Korean L1A strains. (**D**) ML phylogenetic tree of L1C PRRSV-2 sequences showing the distinct clustering of Korean L1C strains and their phylogenetic proximity to the Minnesota17B strain (GenBank: KP283402), rather than to the prototype NADC30 strain (GenBank: JN654459). (**E**) Pairwise ORF5 nucleotide identity (%) between Korean, U.S., and Chinese L1C sequences, as well as within Korean L1C strains

Recombination screening of the complete dataset (*n* = 1,903) using RDP4 (v4.101) detected no statistically supported events in the ORF5 region, indicating that the subsequent phylogenetic clustering patterns were not influenced by recombination. A maximum likelihood (ML) phylogenetic tree was constructed including 907 non-redundant Korean PRRSV-2 ORF5 sequences collected between 2018 and 2024. According to the refined classification by Yim-Im et al. [19], Korean viruses were distributed across multiple lineages, including commercial vaccine-derived clusters (L5A: Ingelvac MLV-like; L8C: Fostera MLV-like), Korean-specific lineages (L1J, L11, and LKB), and globally widespread lineage 1 sublineages (L1A, L1B, and L1C) (Fig. 1A).

Notably, the LKB clade, which has been circulating in Korea since the early 2010s [39, 40], formed a distinct ORF5-defined phylogenetic cluster that was genetically separated from all 11 currently defined lineages. The average pairwise nucleotide distances between LKB and other lineages ranged from 15.63% to 19.85%, exceeding the conventional inter-lineage divergence threshold of approximately 10–12% used for PRRSV-2 lineage discrimination (Supplementary Table 2). Historical Korean LKB isolates, such as GGYC45 (MZ287324; isolated in 2010) and JB15-N-PJ10-GN (MZ287321; isolated in 2015) [32, 50], were included in this cluster, confirming its long-term presence in the field. Based on these findings, the pre-existing LKB clade, which was not incorporated into the recently proposed L11 system [19], was designated as a distinct lineage, proposed here as lineage 12 (L12) (Fig. 1A).

In contrast to the long-term circulation of LKB, the recent increase in prevalence of PRRSV-2 L1 in Korea prompted further examination of potential Korean-adapted lineage 1 subpopulations. To this end, sublineage-specific ML trees were reconstructed for L1A and L1C. Within the L1A-specific tree (Fig. 1B), Korean L1A strains exhibited relatively low ORF5 nucleotide identity with U.S. L1A strains (average 94.0%, range 89.3–96.8%) and Chinese L1A strains (average 92.3%, range 88.4–95.9%), while showing high within-group homology (average 97.1%, range 93.7–100%) (Fig. 1C).

Similarly, in the L1C-specific phylogenetic tree (Fig. 1D), Korean L1C strains clustered into a discrete group, genetically distinct from U.S. and Chinese L1C variants. The Korean L1C group showed high within-group homology (average 96.8%, range 93.5–99.7%) but comparatively lower identity to U.S. L1C strains (average 91.5%, range 86.9–95.2%) (Fig. 1E). Interestingly, these Korean L1C strains were more closely related to the Minnesota17B strain (GenBank accession number KP283402, isolated in 2013) than to the prototype NADC30 strain (GenBank accession number JN654459, isolated in 2008) (Fig. 1D). These Korean L1C strains were phylogenetically divergent from previously defined L1C subclades (L1C.1–L1C.5) [19], forming a unique cluster.

### Spatiotemporal dynamics of PRRSV-2 lineages and emergence of NADC34-like viruses in Korea

**Fig. 2:**
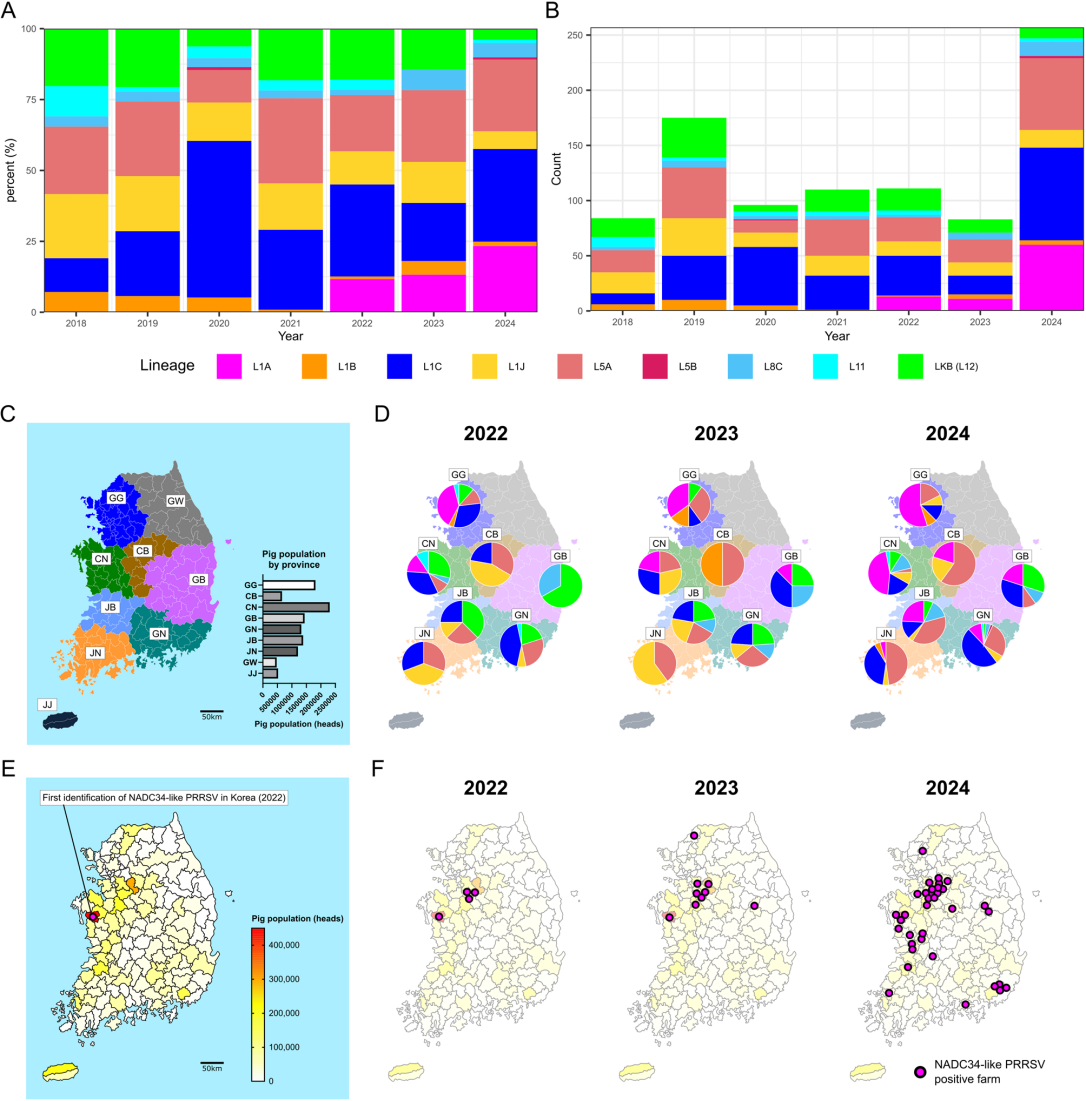
Temporal and spatial dynamics of PRRSV-2 lineages in Korea from 2018 to 2024. (**A**) Proportional distribution and (**B**) absolute number of PRRSV-2 lineages and sublineages identified from field cases between 2018 and 2024. (**C**) Provincial pig population in Korea based on data from the Korean Statistical Information Service (KOSIS; https://kosis.kr/eng/). (**D**) Provincial distribution of PRRSV-2 lineages from 2022 to 2024. (**E**) Location of the first L1A-positive case identified in 2022, with overlaid regional pig density from KOSIS. (**F**) Spatial spread of L1A NADC34-like PRRSV-positive farms across Korea between 2022 and 2024

To investigate recent epidemiological trends of PRRSV-2 in Korea, we analyzed the annual distribution of genetic lineages among field-detected strains between 2018 and 2024 (Fig. 2). Lineage 1 (L1) consistently represented the most dominant genotype throughout the study period, accounting for over 60% of sequenced field cases in 2020. Among the sublineages of L1, L1C (NADC30-like) was predominant from 2018 to 2021, followed by a marked increase in the proportion of L1A (NADC34-like) strains in 2024 (23.3%) since its first detection in 2022 (11.7%) (Fig. 2A and B).

Given the recent emergence and rapid expansion of L1A, we further examined its spatial distribution from 2022 to 2024. In 2022, L1A cases were limited to Gyeonggi-do (GG) and Chungcheongnam-do (CN), both of which are major swine-producing provinces (Fig. 2C and D). This early localization coincided with the location of the first confirmed L1A-positive farm in CN (Fig. 2E). However, by 2023 and 2024, L1A cases became more widespread, with notable increases in both detection frequency and spatial extent across additional provinces (Fig. 2D and F).

### Clinical and production impact of a NADC34-like PRRSV outbreak case in Korea

To evaluate the clinical and productivity impact of the NADC34-like PRRSV outbreak in Korea, we analyzed 38-week production data from a 3,000-sow farrow-to-weaning farm (Farm A) that experienced an outbreak in May 2023. The dataset was divided into pre-outbreak (19 weeks) and post-outbreak (19 weeks) periods. The causative PRRSV-2 strain isolated during the outbreak, designated JBNU-23-N05 (GenBank accession number: PV459697), shared 98.4% nucleotide identity at the whole-genome level and 98.3% identity in the ORF5 gene with JBNU-22-N01 (GenBank accession number: OP970983), the first NADC34-like PRRSV-2 isolated in Korea in 2022 [37]. Detailed nucleotide and amino acid similarities across the full genome, compared with JBNU-22-N01, NADC34 (L1A reference), and the RespPRRS MLV vaccine strain, are summarized in Table 1.

**Table 1:** Nucleotide and amino acid similarities of the Korean NADC34-like isolate JBNU-23-N05 (Farm A, Korea, 2023 outbreak) with the first Korean L1A isolate JBNU-22-N01, the L1A prototype NADC34 strain, and the RespPRRS MLV vaccine strain. Values are shown as nucleotide % identity / amino acid % identity for each ORF or protein

	JBNU-22-N01 (OP970983)	IA/2014/NADC34 (MF326985)	RespPRRS-MLV (AF066183)
**Nucleotides**			
Complete genome	98.4%	89.7%	82.2%
ORF1a	98.2%	86.1%	79.3%
ORF1b	98.6%	90.3%	84.1%
ORF2-7	98.4%	95.9%	85.3%
**Nucleotides / amino acids**			
nsp1α	97.5% / 97.8%	92.1% / 95.5%	86.7% / 95.5%
nsp1β	98.3% / 97.0%	87.6% / 86.2%	75.2% / 74.2%
nsp2	97.7% / 96.6%	86.1% / 84.7%	72.0% / 66.3%
nsp3	99.0% / 100%	85.7% / 92.8%	83.1% / 94.2%
nsp4	99.2% / 99.0%	79.0% / 90.8%	91.5% / 93.4%
nsp5	98.6% / 97.6%	81.4% / 87.5%	94.0% / 92%
nsp6	97.8% / 100%	90.8% / 93.5%	95.2% / 100%
nsp7α	97.9% / 98.6%	86.6% / 96.6%	79.4% / 92.3%
nsp7β	99.1% / 99.1%	87.6% / 91.5%	74.9% / 81.0%
nsp8	100% / 100%	93.7% / 93.1%	86.8% / 85.7%
nsp9	98.3% / 98.9%	91.1% / 97.1%	85.4% / 95.9%
nsp10	98.7% / 99.3%	88.6% / 98.7%	82.9% / 94.4%
nsp11	98.9% / 99.1%	90.1% / 94.0%	84.1% / 94.0%
nsp12	98.9% / 100%	90.5% / 96.7%	82.0% / 90.4%
GP2	98.6% / 97.6%	97.2% / 97.6%	87.3% / 86.6%
GP3	98.3% / 97.6%	95.2% / 95.6%	81.6% / 81.0%
GP4	98.5% / 98.9%	95.5% / 97.7%	84.5% / 86.8%
GP5	98.3% / 98.0%	95.9% / 95.4%	86.0% / 85.5%
M	98.6% / 98.3%	97.1% / 98.3%	86.8% / 92.9%
N	98.1% / 99.2%	94.3% / 95.9%	86.9% / 92.4%

**Fig. 3:**
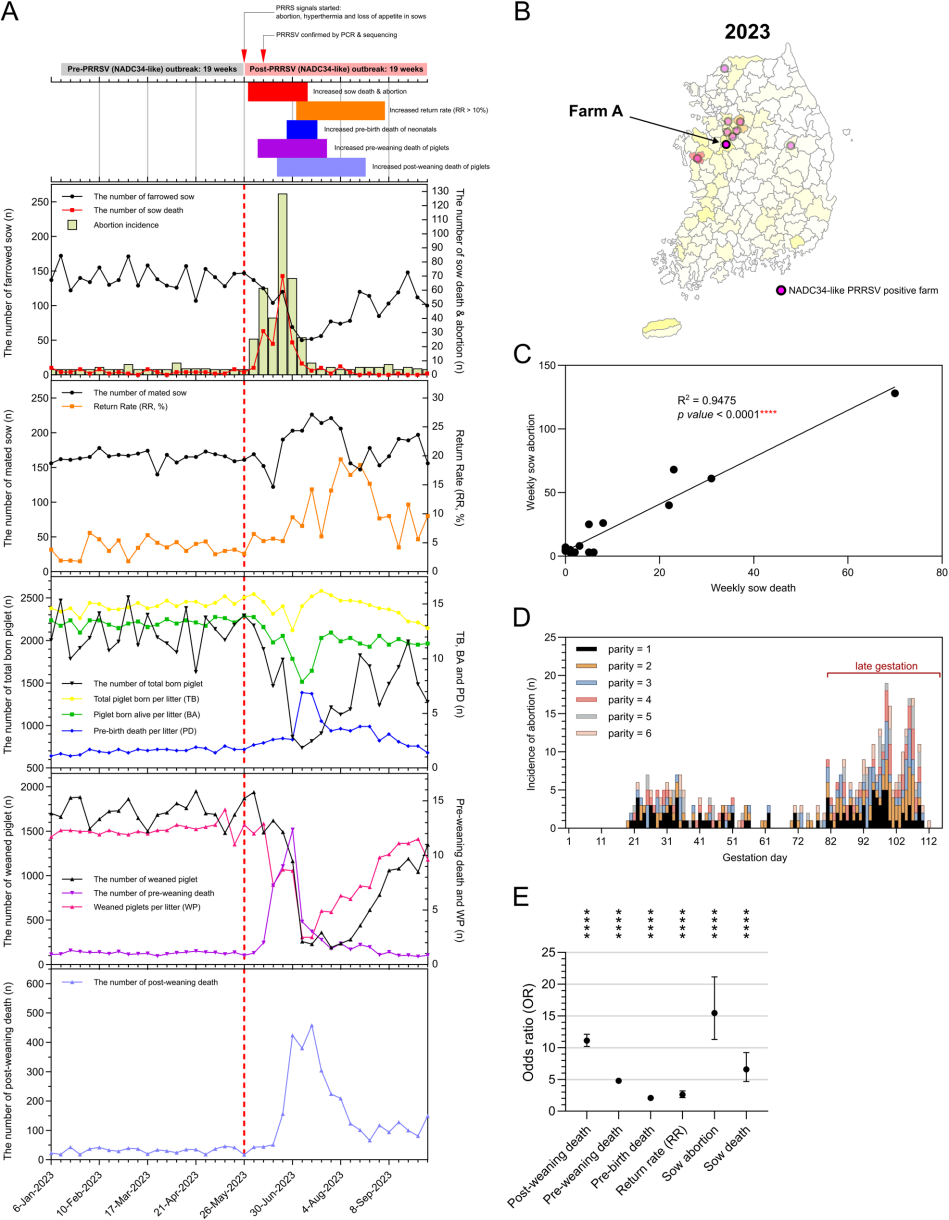
Farm-level production impact following a Korean NADC34-like PRRSV-2 outbreak. (**A**) Weekly production trends from a 3,000-sow farrow-to-weaning farm during 19-week pre- and post-outbreak periods. The top timeline summarizes major outbreak-related events. Five production parameters are displayed across panels: (1) weekly sow abortions and deaths, (2) return-to-estrus rate (RR), (3) reproductive performance (pre-birth mortality per parity), (4) pre-weaning piglet mortality, and (5) post-weaning piglet mortality. (**B**) Geographic location of Farm A in Chungnam Province, Korea. (**C**) Positive correlation between weekly abortion and sow mortality rates during the outbreak period. (**D**) Distribution of gestational age at abortion among affected sows. (**E**) Odds ratios with 95% confidence intervals comparing pre- and post-outbreak periods across six productivity indicators. Asterisks denote statistical significance (**** indicates *p*-values < 0.0001)

The outbreak was initially characterized by fever and reduced feed intake in sows, followed by severe productivity losses across all stages, including pregnant sows, neonatal piglets, weaned piglets, and re-bred sows after abortion or weaning (Fig. 3). During the first six weeks of the outbreak, a total of 411 out of 1,990 sows (20.7%) aborted, and 181 sows (9.1%) died, compared to 46 abortions (1.6%) and 42 deaths (1.5%) among 2,810 sows in the pre-outbreak period (Fig. 3A, panel 1). A significant positive correlation was observed between weekly sow abortion and mortality rates (*p* < 0.0001) (Fig. 3C). Most abortions occurred during late gestation (77–115 days of gestation), comprising 64.7% (266/411) of all cases, followed by the early- (0–38 days, 20.7%) and mid-gestation (39–76 days, 14.6%) (Fig. 3D).

Surviving infected sows that did not abort showed an increased return-to-estrus rate (RR), defined as inseminated sows failing to establish pregnancy and subsequently returning to estrus, which reached 20% post-insemination, compared with < 5% before the outbreak (Fig. 3A, panel 2). Fetal loss among infected sows also increased, with pre-birth deaths, defined as the difference between total piglets born (TB) and piglets born alive (BA), and therefore including both stillbirths and mummified fetuses, averaging up to 6.9 piglets per sow, compared to 1.5 pre-outbreak (Fig. 3A, panel 3).

Neonatal mortality during the first six weeks surged dramatically, reaching a peak of 1,518 deaths in one week versus a pre-outbreak average of 136.8 per week, corresponding to a pre-weaning mortality rate of 23.3% (6,780/29,160) compared with 6.2% (2,600/41,614) in the pre-outbreak period (Fig. 3A, panel 4). Post-weaning mortality similarly rose to 458 piglets per week (17.5%, 3,269/18,694), compared with an average 31.5 piglets per week (1.9%, 640/34,238) before the outbreak (Fig. 3A, panel 5).

Odds ratio analysis comparing pre- and post-outbreak periods confirmed a significantly elevated risk for all metrics: sow mortality (OR 6.59, 95% CI 4.68–9.24), abortion (15.45, 11.30–21.15), return to estrus (2.44, 2.02–2.96), pre-birth death (2.07, 1.98–2.16), pre-weaning mortality (4.78, 4.55–5.01), and post-weaning mortality (11.12, 10.19–12.13) (Fig. 3E). While post-weaning mortality exhibited a notably high odds ratio, its increase occurred with a temporal lag of approximately three weeks following the initial rise in sow abortions (Fig. 3A, panel 5). This delay suggests that elevated post-weaning mortality was not the result of direct exposure to PRRSV during the nursery phase, but rather a downstream consequence of prenatal or neonatal exposure. Cross-correlation analysis (Supplementary Fig. 1) further supported this pattern, showing the strongest association between abortion and post-weaning mortality at a three-week lag.

### Pathological findings among affected sows from NADC34-like outbreak cases

As shown in Supplementary Table 3, isolates from Farms A–C clustered closely within the NADC34-like (L1A) group in ORF5 gene level (96.9–98.8% nucleotide similarity), supporting their genetic relatedness despite being derived from independent outbreaks. To further characterize their pathogenic impact, postmortem examinations were performed on sows that died during confirmed NADC34-like outbreaks at Farm B (Gyeonggi-do, March 2023) and Farm C (Chungcheongbuk-do, March 2024).

**Fig. 4:**
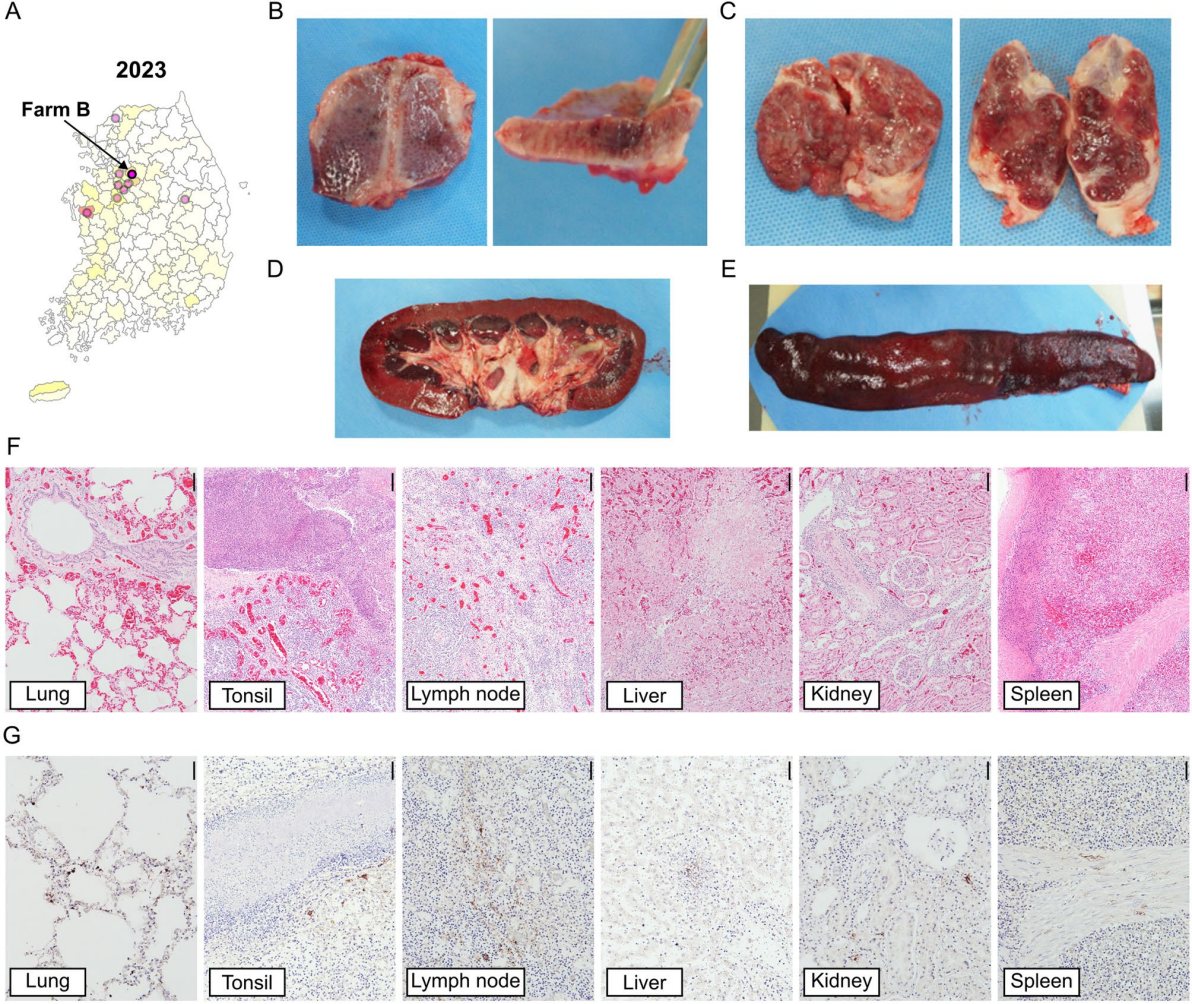
Post-mortem pathological findings from a sow affected by NADC34-like PRRSV in Farm B (March 2023). (**A**) Geographic location of Farm B in Gyeonggi Province, Korea. (**B**) Purple red discoloration (left) and formation of dark red round lesion in the tonsil (cut surface, right). (**C**) Enlarged lymph nodes. (**D**) Dark red discoloration in renal corticomedullary junction (cut surface of the kidney). (**E**) Enlarged spleen with dull edges and dark red discoloration. (**F**) Histopathologic features in various internal organs (H&E, scale bar = 100 μm): lung – diffuse pulmonary congestion and edema; tonsil – focal bacterial abscess with adjacent marked congestion; LN – reactive lymphoid hyperplasia with multifocal necrotizing lymphadenitis; liver – marked multifocal necrotizing hepatitis with centrilobular congestion; kidney – multifocal interstitial nephritis with non-suppurative periarteritis; spleen – partial necrosis and hemorrhage in the red pulp. (**G**) Immunohistochemical detection of PRRSV antigens in various organs. Scale bar = 50 μm

**Fig. 5:**
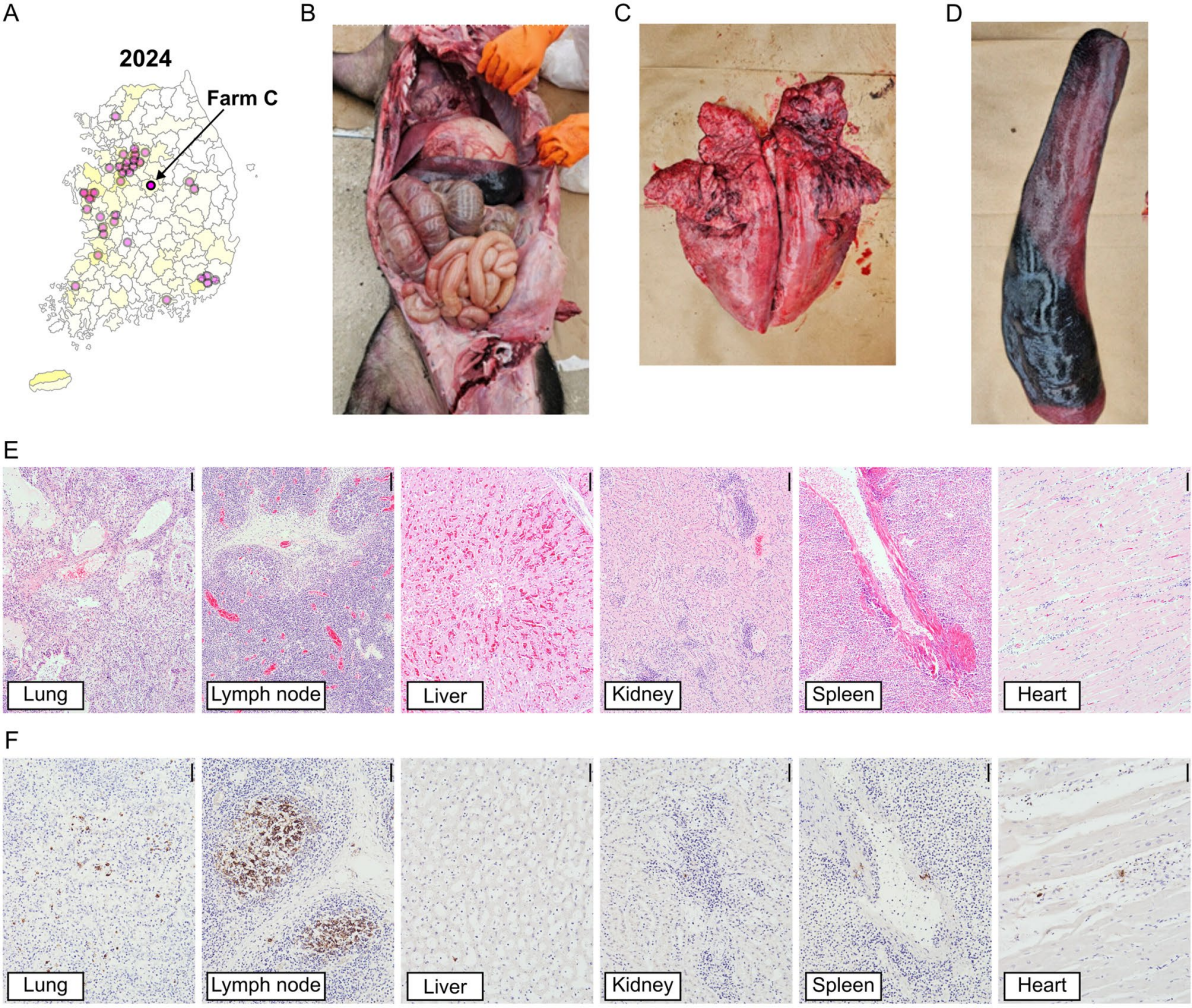
Post-mortem pathological findings from a gilt affected by NADC34-like PRRSV in Farm C (March 2024). (**A**) Geographic location of Farm C in Chungbuk Province, Korea. (**B**) Serohemorrhagic effusion in the thoracic cavity. (**C**) Diffuse bright red discoloration with dark red lobular consolidation in the cranioventral lobes. (**D**) Enlarged spleen with dark red appearance. (**E**) Histopathologic features in various internal organs (H&E, scale bar = 100 μm): lung – diffuse bronchointerstitial pneumonia with marked pulmonary vasculitis; LN – reactive lymphoid hyperplasia; liver – portal inflammatory cell infiltration with centrilobular congestion; kidney – multifocal interstitial nephritis with non-suppurative periarteritis; spleen – Vasculitis in the trabecular blood vessels; heart – multifocal non-suppurative myocarditis. (**F**) Immunohistochemical detection of PRRSV antigens in various organs. Scale bar = 50 μm

Across both outbreaks, major gross lesions consistently included generalized enlargement of lymph nodes, splenomegaly, and diffuse pulmonary congestion with multifocal cranioventral consolidation. Microscopic examination revealed the histopathologic patterns of systemic infection characterized by circulatory disorder, lymphoid hyperplasia, and multifocal necrosis affecting multiple organs. Notably, both cases showed interlobular septal edema with vasculitis or bronchopneumonia in the lung, reactive lymphoid hyperplasia with multifocal necrotizing lymphadenitis, necrotizing hepatitis, multifocal interstitial nephritis with periarteritis of medium-to-large renal arteries, multifocal splenic necrosis and vasculitis, and non-suppurative myocarditis (Figs. 4 and 5).

Overall, these findings indicate that Korean NADC34-like PRRSV variants were associated with systemic vasculitis and multi-organ lesions, extending beyond the classical pulmonary–reproductive tropism typically ascribed to other PRRSV strains. The recurrence of these non-conventional lesions across independent farms suggests a potential shift in virulence or tissue tropism specific to Korean L1A field variants. PCR/RT-PCR testing for differential diagnosis of other systemic infections was negative for all tested pathogens in both cases.

## Discussion

PRRSV-2 exhibits extensive genetic variability [27, 51], and its phylogenetic classification has been refined multiple times to reflect viral evolution and expanding sequence datasets [19, 52]. The most recent framework by Yim-Im et al. (2023), based on over 82,000 ORF5 sequences, defined 11 lineages and 21 sublineages, and incorporated several Korean-origin groups such as LKA and LKC as L11 and L1J, respectively [19, 39, 40, 43]. However, the Korean-specific LKB clade, first detected in the early 2010s and still circulating [39, 40, 53], was not included in this system. At the same time, the viral landscape has become increasingly diverse due to the introduction and spread of globally circulating L1 sublineages, including L1C [39], L1B [40], and most recently L1A [37], further underscore the need to reevaluate and refine lineage classification frameworks for PRRSV-2 in Korea, particularly in the context of both global and local evolutionary dynamics.

In this study, we analyzed 907 non-redundant Korean PRRSV-2 ORF5 sequences collected between 2018 and 2024, applying the updated classification framework for consistency and comparability [19]. Phylogenetic analysis demonstrated that LKB strains formed a distinct ORF5-defined cluster, genetically separated from the 11 recognized lineages (Fig. 1A). Pairwise nucleotide distance analysis further supported this distinction, showing 15.63–19.85% divergence from other lineages (Supplementary Table 2), well above the 10–12% threshold typically used for lineage designation. Based on these results, we propose the formal recognition of LKB as Lineage 12 (L12) within the PRRSV-2 classification framework.

The evolutionary origin of L12 remains under investigation. Our previous whole-genome study suggested that LKB may have originated through inter-lineage recombination between the Korean-specific LKC sublineage and vaccine-derived L5 strains [43]. Since its emergence, however, the LKB/L12 group has maintained a distinct and stable ORF5-defined phylogenetic signature, with isolates detected consistently since the early 2010s [39, 40], and showing somewhat higher pathogenicity under experimental conditions compared to other lineages/isolates [21, 50, 54]. Although no recombination events were detected within ORF5 in the present dataset, recombination at the whole-genome level can generate mosaic viruses, potentially challenging the long-term validity of ORF5-based lineage assignments. Thus, while ORF5-based classification provides a practical framework for short-term epidemiological surveillance, its conceptual and practical limitations must be acknowledged. Complementary whole-genome sequencing will be essential for fully understanding the evolutionary dynamics of L12 and other emerging PRRSV-2 variants.

Geographical variation in PRRSV-2 diversity, both in terms of genetic heterogeneity and the duration of lineage/sublineage circulation, has been widely recognized [19]. In this study, Korean L1C and L1A strains formed distinct ORF5-defined clusters clearly separated from their U.S. and Chinese counterparts (Fig. 1B and E). These clusters were characterized by high intra-cluster nucleotide identity and lower similarity to foreign strains, suggesting independent regional diversification after introduction. This pattern is consistent with the previously described evolutionary trajectories of Chinese NADC30-like (L1C) strains following introduction [31]. Notably, Korean L1C strains clustered more closely with an earlier North American strain (Minnesota17B, isolated in 2013) than with the prototype NADC30 strain (2008), and were genetically distinct from the five L1C subclades (L1C.1–L1C.5) that are currently predominant in North America. Although the precise routes of viral introduction remain unclear, our findings emphasize the importance of continuous surveillance and strengthened biosecurity to mitigate further diversification and dissemination of PRRSV-2.

Following its first detection in 2022 [37], L1A rapidly expanded across Korea, gradually dominating PRRSV-2 lineages among field cases by 2024 (Fig. 2A and B), which was similar to the earlier expansion of L1C within Korea [40]. Initially concentrated in the central-western provinces (Gyeonggi-do and Chungcheongnam-do), L1A spread extensively across multiple regions by 2024 (Fig. 2C and F), indicating successful establishment and inter-regional dissemination.

The widespread dissemination of L1A raises particular concern given its well-documented association with severe clinical outbreaks in North America. In the U.S., L1A has been implicated in “abortion storms” in sow herds and marked increases in neonatal piglet mortality [33, 34], often leading to substantial productivity losses and prolonged recovery periods on affected farms. Similar clinical manifestations were observed in the Korean field case described in this study (Fig. 3). The outbreak caused by a Korean L1A strain in May 2023 resulted in a cascade of reproductive failures, including extensively increased abortion incidences, and fetal loss, followed by a sharp rise in pre- and post-weaning mortality (Fig. 3A). Notably, this outbreak occurred despite routine vaccination with a commercial PRRSV-2 MLV vaccine, consistent with previous reports of NADC34-like outbreaks in vaccinated herds [37, 55], and with similar outcomes confirmed in this study (Farms B and C). Considering that no MLV vaccine is currently based on L1A strains, these findings suggest that existing vaccines may offer limited cross-protection against emerging NADC34-like variants, highlighting the urgent need for updated or strain-specific vaccine formulations.

Importantly, a particularly striking feature of the Korean outbreak was the extensive sow mortality, a clinical manifestation that has been rarely reported in L1A-related outbreaks in North America [33, 34] or China [29, 56]. Within six weeks post-outbreak, nearly 10% of sows in the affected herd had died, with sow deaths occurring in close parallel with abortion events (Fig. 3A and C). Supporting this observation, additional pathological investigations from other NADC34-like PRRSV outbreaks in Korea (Farm B in 2023 and Farm C in 2024) confirmed a consistent systemic lesion pattern, notably involving vasculitis, necrosis, and lymphoid hyperplasia across multiple organs (Figs. 4 and 5). The recurrence of these systemic lesions across two independent outbreak cases suggests that the Korean NADC34-like PRRSV variant may exhibit enhanced systemic pathogenicity compared to previously described L1A-associated pathology, which has largely been limited to reproductive or respiratory involvement [29, 33, 34, 56]. This expanded pathogenic profile highlights the need for further genomic and immunopathological studies to determine whether Korean strains have evolved increased virulence or novel tissue tropism.

Another notable feature of the NADC34-like PRRSV outbreak was the delayed increase in post-weaning mortality, which peaked about three weeks after the initial surge in abortions (Supplementary Fig. 1). This pattern suggests an indirect consequence of prenatal or early-life viral exposure rather than acute infection in the nursery phase. Although direct infection rate data at weaning were not available, our previous in vivo challenge study using the Korean NADC34-like strain JBNU-22-N01 showed that clinical severity in weaned piglets was comparable to VR2332 and NADC30-like (L1C) strains, with no mortality observed [57]. These findings, together with the low initial post-weaning mortality observed in the present outbreak, indicate that nursery pigs present before the outbreak were not critically affected. Nevertheless, further studies are warranted to clarify the age-dependent pathogenicity of NADC34-like PRRSV and to determine whether prenatal or early-life exposure may predispose nursery pigs to later health challenges.

## Conclusions

This study provides a comprehensive analysis of the evolving genetic and clinical landscape of PRRSV-2 in Korea. We identified a distinct Korean-specific lineage, L12, and proposed its formal designation based on phylogenetic divergence, highlighting the need to update existing classification systems. Our findings also demonstrate that globally prevalent L1 sublineages, particularly NADC34-like PRRSV (L1A), have been repeatedly introduced into Korea, followed by local diversification and spread. Notably, the Korean NADC34-like outbreak exhibited atypical clinical features, including sow mortality and delayed post-weaning losses, despite routine vaccination. These observations raise concerns regarding vaccine cross-protection and suggest that viral recombination and immunomodulation may contribute to altered pathogenicity. Ongoing genomic surveillance, functional studies, and the development of strain-adapted vaccines are essential to mitigate the risks posed by emerging PRRSV-2 variants. Strengthened international collaboration and biosecurity measures will further support these efforts.

## Supplementary Information

Below is the link to the electronic supplementary material.

Supplementary Material 1

## Data Availability

The data that support the findings of this study are available from the corresponding author upon reasonable request.
