# Supplementary material for: Nationwide emergence and spread of highly virulent PRRSV-2 mutants in Korea
Source: Porcine Health Manag. 2025 Nov 11;11:58. doi: 10.1186/s40813-025-00470-5 (PMC12606797; doi:10.1186/s40813-025-00470-5)
Supplement: Supplementary file 1 — Supplementary Material 1 [file 40813_2025_470_MOESM1_ESM.docx]

**Supplementary Table 1. Annual and regional distribution of Korean PRRSV-2 ORF5 sequences included in this study (2018–2024). Sequences were obtained from clinical samples submitted to the Jeonbuk National University Veterinary Diagnostic Center (JBNU-VDC), Kyungpook National University (KNU), and from GenBank (NCBI). To minimize redundancy, a total of 1305 sequences were subsampled using CD-HIT at a 99.9% similarity threshold. Numbers represent the counts of non-redundant sequences per year and geographic region: GG (Gyeonggi), CB (Chungbuk), CN (Chungnam), GB (Gyeongbuk), GN (Gyeongnam), JB (Jeonbuk), JN (Jeonnam), GW (Gangwon), JJ (Jeju), and Unknown (region not available). The merged dataset, covering all major swine-producing regions of Korea, was used to generate the final non-redundant panel of 907 ORF5 sequences.**

| JBNU-VDC | Region | 2018 | 2019 | 2020 | 2021 | 2022 | 2023 | 2024 | Sum |
| --- | --- | --- | --- | --- | --- | --- | --- | --- | --- |
|  | **GG** | 0 | 2 | 0 | 16 | 26 | 20 | 40 | 104 |
|  | **CB** | 1 | 0 | 0 | 4 | 9 | 2 | 5 | 21 |
|  | **CN** | 0 | 0 | 0 | 11 | 21 | 14 | 33 | 79 |
|  | **GB** | 1 | 0 | 0 | 4 | 3 | 8 | 10 | 26 |
|  | **GN** | 1 | 84 | 1 | 33 | 30 | 25 | 116 | 290 |
|  | **JB** | 1 | 0 | 0 | 9 | 8 | 9 | 29 | 56 |
|  | **JN** | 2 | 0 | 0 | 6 | 13 | 5 | 23 | 49 |
|  | **GW** | 0 | 0 | 0 | 0 | 0 | 0 | 0 | 0 |
|  | **JJ** | 0 | 0 | 0 | 0 | 0 | 0 | 0 | 0 |
|  | **Unknown** | 60 | 17 | 0 | 0 | 0 | 0 | 0 | 77 |
|  | **Sum** | 66 | 103 | 1 | 83 | 110 | 83 | 256 | 702 |
| KNU | **Region** | **2018** | **2019** | **2020** | **2021** | **2022** | **2023** | **2024** | **Sum** |
|  | **GG** | 3 | 17 | 33 | 8 | 0 | 0 | 0 | 61 |
|  | **CB** | 0 | 4 | 9 | 1 | 0 | 0 | 0 | 14 |
|  | **CN** | 0 | 11 | 8 | 0 | 0 | 0 | 0 | 19 |
|  | **GB** | 4 | 15 | 11 | 5 | 0 | 0 | 0 | 35 |
|  | **GN** | 1 | 6 | 14 | 6 | 0 | 0 | 0 | 27 |
|  | **JB** | 0 | 6 | 4 | 0 | 0 | 0 | 0 | 10 |
|  | **JN** | 0 | 4 | 5 | 1 | 0 | 0 | 0 | 10 |
|  | **GW** | 3 | 2 | 3 | 1 | 0 | 0 | 0 | 9 |
|  | **JJ** | 0 | 6 | 6 | 2 | 0 | 0 | 0 | 14 |
|  | **Unknown** | 0 | 0 | 0 | 0 | 0 | 0 | 0 | 0 |
|  | **Sum** | 11 | 71 | 93 | 24 | 0 | 0 | 0 | 199 |
| NCBI | **Region** | **2018** | **2019** | **2020** | **2021** | **2022** | **2023** | **2024** | **Sum** |
|  | **GG** | 0 | 0 | 0 | 0 | 0 | 0 | 0 | 0 |
|  | **CB** | 0 | 0 | 0 | 0 | 0 | 0 | 0 | 0 |
|  | **CN** | 0 | 0 | 0 | 0 | 0 | 0 | 0 | 0 |
|  | **GB** | 0 | 0 | 0 | 0 | 0 | 0 | 0 | 0 |
|  | **GN** | 0 | 0 | 0 | 0 | 0 | 0 | 0 | 0 |
|  | **JB** | 0 | 0 | 0 | 0 | 0 | 0 | 0 | 0 |
|  | **JN** | 0 | 0 | 0 | 0 | 0 | 0 | 0 | 0 |
|  | **GW** | 0 | 0 | 0 | 0 | 0 | 0 | 0 | 0 |
|  | **JJ** | 0 | 0 | 0 | 0 | 0 | 0 | 0 | 0 |
|  | **Unknown** | 3 | 0 | 2 | 0 | 1 | 0 | 0 | 6 |
|  | **Sum** | 3 | 0 | 2 | 0 | 1 | 0 | 0 | 6 |
| Merged (JBNU-VDC + KNU + NCBI) | **Region** | **2018** | **2019** | **2020** | **2021** | **2022** | **2023** | **2024** | **Sum** |
|  | **GG** | 3 | 19 | 33 | 24 | 26 | 20 | 40 | 165 |
|  | **CB** | 1 | 4 | 9 | 5 | 9 | 2 | 5 | 35 |
|  | **CN** | 0 | 11 | 8 | 11 | 21 | 14 | 33 | 98 |
|  | **GB** | 5 | 15 | 11 | 9 | 3 | 8 | 10 | 61 |
|  | **GN** | 2 | 90 | 15 | 39 | 30 | 25 | 116 | 317 |
|  | **JB** | 1 | 6 | 4 | 9 | 8 | 9 | 29 | 66 |
|  | **JN** | 2 | 4 | 5 | 7 | 13 | 5 | 23 | 59 |
|  | **GW** | 3 | 2 | 3 | 1 | 0 | 0 | 0 | 9 |
|  | **JJ** | 0 | 6 | 6 | 2 | 0 | 0 | 0 | 14 |
|  | **Unknown** | 63 | 17 | 2 | 0 | 1 | 0 | 0 | 83 |
|  | **Sum** | 80 | 174 | 96 | 107 | 111 | 83 | 256 | 907 |

**Supplementary Table 2. Average pairwise nucleotide differences (%) among PRRSV-2 ORF5 sequences classified into 12 lineages, including 11 previously defined lineages (Yim-Im et al., 2023) and the proposed lineage L12 (formerly LKB).**

| **Lineage** | **L1** | **L2** | **L3** | **L4** | **L5** | **L6** | **L7** | **L8** | **L9** | **L10** | **L11** | **L12 (LKB)** |
| --- | --- | --- | --- | --- | --- | --- | --- | --- | --- | --- | --- | --- |
| **L1** | 14.49 |  |  |  |  |  |  |  |  |  |  |  |
| **L2** | 18.77 | 11.14 |  |  |  |  |  |  |  |  |  |  |
| **L3** | 20.15 | 19.94 | 14.06 |  |  |  |  |  |  |  |  |  |
| **L4** | 16.70 | 15.13 | 16.96 | 9.72 |  |  |  |  |  |  |  |  |
| **L5** | 17.82 | 13.77 | 18.25 | 12.85 | 4.82 |  |  |  |  |  |  |  |
| **L6** | 18.86 | 17.30 | 20.73 | 15.45 | 13.39 | 5.78 |  |  |  |  |  |  |
| **L7** | 16.72 | 14.19 | 16.79 | 10.75 | 10.59 | 13.25 | 0.36 |  |  |  |  |  |
| **L8** | 18.42 | 16.23 | 18.68 | 14.10 | 13.43 | 14.85 | 11.45 | 10.53 |  |  |  |  |
| **L9** | 18.38 | 16.14 | 18.87 | 13.79 | 13.47 | 14.00 | 10.44 | 12.62 | 10.56 |  |  |  |
| **L10** | 17.60 | 14.88 | 18.09 | 13.14 | 12.94 | 15.82 | 10.29 | 13.29 | 12.94 | 4.52 |  |  |
| **L11** | 18.27 | 16.65 | 18.65 | 15.39 | 15.15 | 17.07 | 15.01 | 16.85 | 16.13 | 15.70 | 12.37 |  |
| **L12 (LKB)** | 19.85 | 18.58 | 19.64 | 17.00 | 15.63 | 18.41 | 15.83 | 17.44 | 17.85 | 17.25 | 18.48 | 11.61 |

**Supplementary Table 3. Pairwise nucleotide and amino acid similarities of ORF5 sequences among NADC34 (MF326985), the first Korean NADC34-like isolate JBNU-22-N01 (OP970983, 2022), the Farm A outbreak strain JBNU-23-N05 (PV459697, 2023), and field isolates from Farm B (PV425837, 2023) and Farm C (PV425849, 2024). Values are presented as nucleotide % identity / amino acid % identity.**

| Nucleotide / amino acid similarity | NADC34 | JBNU-22-N01 | JBNU-23-N05 | JBNU-VDC-2023-001 | JBNU-VDC-2024-001 |
| --- | --- | --- | --- | --- | --- |
| MF326985_NADC34\|2014 |  |  |  |  |  |
| OP970983_JBNU-22-N01\|2022 | 96.6% / 97.5% |  |  |  |  |
| (Farm A) PV459697_JBNU-23-N05\|2023 | 95.9% / 95.4% | 98.3% / 98.0% |  |  |  |
| (Farm B) PV425837_JBNU-VDC-2023-001\|2023 | 96.2% / 97.0% | 98.1% / 99.0% | 97.1% / 97.0% |  |  |
| (Farm C) PV425849_JBNU-VDC-2024-001\|2024 | 95.5% / 96.4% | 97.6% / 99.0% | 96.9% / 98.0% | 98.8% / 98.0% |  |

**
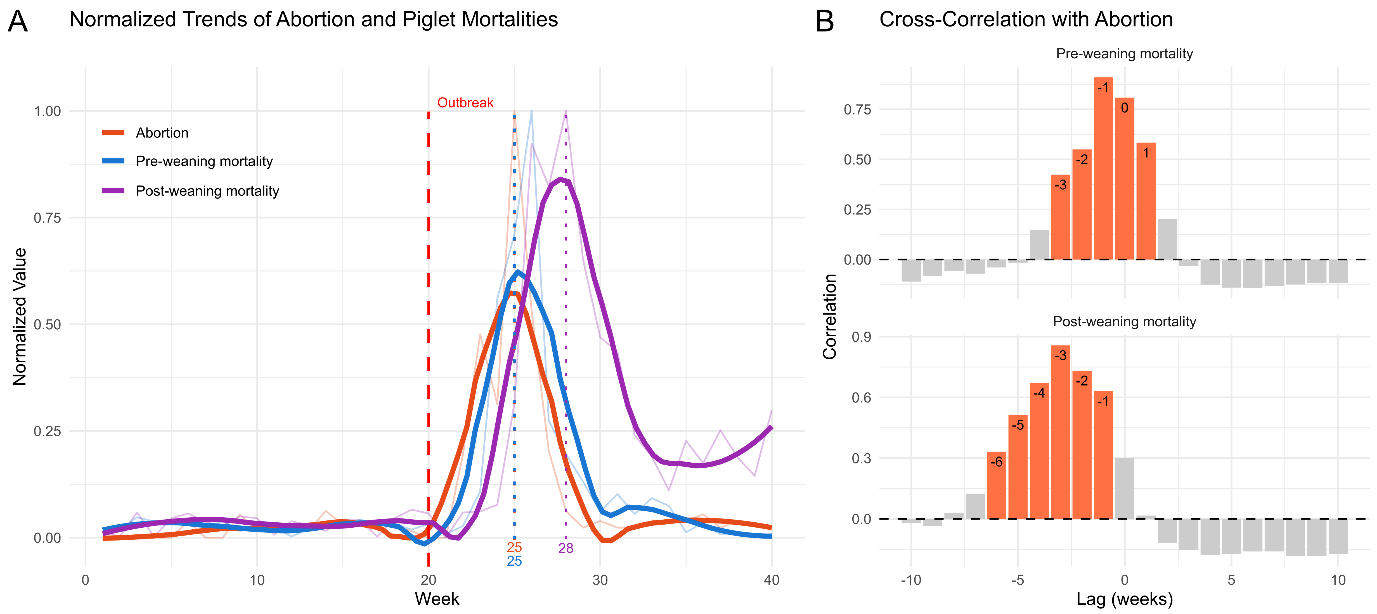
**

**Supplementary Figure 1.** **Temporal relationship between abortion and piglet mortalities following NADC34-like PRRSV outbreak.**
(A) Weekly normalized trends of abortion, pre-weaning mortality, and post-weaning mortality over a 40-week observation period from a 3,000-sow farm that experienced an NADC34-like PRRSV outbreak in week 20 (red dashed line). Colored dotted lines indicate the peak week for each smoothed metric. (B) Cross-correlation analysis between abortion and pre-/post-weaning mortalities. Bars represent correlation coefficients at each time lag (weeks), with orange indicating statistically significant correlations (|r| > 0.31, p < 0.05). The results suggest that pre-weaning mortality followed the abortion peak with minimal delay, while post-weaning mortality showed a lag of 3 weeks, consistent with a delayed impact on piglets born from affected sows.
